# Nanotheranostics in Breast Cancer Bone Metastasis: Advanced Research Progress and Future Perspectives

**DOI:** 10.3390/pharmaceutics16121491

**Published:** 2024-11-21

**Authors:** Lin Miao, Yidan Zhu, Hong Chang, Xinfeng Zhang

**Affiliations:** 1 Department of Breast Surgery, Cancer Hospital of China Medical University, Cancer Hospital of Dalian University of Technology, Liaoning Cancer Hospital and Institute, Shenyang 110042, China; miaolin712@126.com (L.M.); vicky.zyd0823@gmail.com (Y.Z.); 2 Graduate School, China Medical University, Shenyang 110122, China; 3 Department of General Thoracic Surgery and Breast and Endocrinological Surgery, Graduate School of Medicine Dentistry and Pharmaceutical Sciences, Okayama University, Okayama 700-8530, Japan

**Keywords:** drug delivery systems, nanotheranostics, breast cancer, bone metastasis, targeted therapy

## Abstract

Breast cancer is the leading cause of cancer-related morbidity and mortality among women worldwide, with bone being the most common site of all metastatic breast cancer. Bone metastases are often associated with pain and skeletal-related events (SREs), indicating poor prognosis and poor quality of life. Most current therapies for breast cancer bone metastasis primarily serve palliative purposes, focusing on pain management, mitigating the risk of bone-related complications, and inhibiting tumor progression. The emergence of nanodelivery systems offers novel insights and potential solutions for the diagnosis and treatment of breast cancer-related bone metastasis. This article reviews the recent advancements and innovative applications of nanodrug delivery systems in the context of breast cancer bone metastasis and explores future directions in nanotheranostics.

## 1. Introduction

According to Globocan, breast cancer (BC) has become the most commonly diagnosed cancer in 2020 and its incidence and mortality rates are predicted to steadily increase. Female BC was the second leading cause of cancer incidence worldwide in 2022, with an estimated 2.3 million new cases, accounting for 11.6% of all cancer cases. BC is the most common malignancy in women and one of the leading causes of cancer deaths worldwide [1,2]. Advances in diagnostic methodologies for BC have led to more timely detection, facilitated by enhanced screening and physical examination techniques. A growing number of researchers and clinicians are collaborating to refine surgical methods, improve advanced drug delivery systems, and develop innovative therapeutic approaches, all aimed at personalizing treatment plans and increasing the precision of breast cancer therapies. The diagnostic and therapeutic paradigms for BC continue to evolve, receiving positive feedback, with corresponding improvements in patient survival and quality of life. However, the prognosis for metastatic breast cancer remains grim, as metastasis is a leading cause of high mortality in these patients. Common metastatic sites include the bone, liver, lungs, and brain [3]. Skeletal-related events (SREs), such as tumor-induced pathological fractures and spinal cord compression, significantly impair the quality of life and decrease survival rates among patients with bone metastases [4].

Bone metastasis is a clinical complication of patients with advanced breast cancer, which seriously affects the quality of life of patients. Early detection of metastasis is essential for effective treatment. In clinical practice, we currently detect bone metastases from breast cancer by standard imaging methods such as X-ray, bone scintigraphy, computed tomography (CT), and magnetic resonance imaging (MRI). However, the sensitivity to detect bone metastases using traditional imaging modalities is often unable to detect minute lesions in the metastatic bone and negligible cancer-induced osteolysis [5]. The primary treatment of metastatic breast cancer (MBC) is to prolong life and relieve symptoms [6]. Most existing therapies are predominantly palliative, concentrating on pain management, mitigating the risk of SREs, and inhibiting tumor progression [7]. Present treatment options for breast cancer bone metastasis (BCBM) include local therapies (surgery and radiotherapy) and systemic therapies (chemotherapy, targeted therapy, and agents aimed at mitigating bone metastasis and destruction). Bisphosphonates (BPs), which inhibit osteoclast differentiation, bone adhesion, and the production and secretion of lyase, are commonly used in treating malignant tumor bone metastases. However, their clinical use is often limited by side effects, including hypercalcemia and renal dysfunction. Commonly used BPs such as alendronic acid and zoledronic acid work by inactivating osteoclasts, simultaneously providing adjunctive therapeutic effects for SREs—such as enhancing bone mineral density and reducing fracture risk, thereby alleviating pain at metastatic sites [8]. A significant challenge in the treatment of metastatic breast cancer is that many antineoplastic agents are cytotoxic. If not selectively delivered to metastatic sites, these agents can cause severe nephrotoxicity, hepatotoxicity, and adverse effects on healthy organs. Furthermore, the complex structure of the bony sinuses and slow local blood flow hinder the accumulation of locally administered chemotherapeutics, resulting in suboptimal biodistribution of intravenous anticancer agents within the bone. The dense bone matrix and unique microenvironment further complicate drug penetration and accumulation at metastatic sites, leading to several issues with conventional drug therapies, including difficulties in achieving targeted delivery, high toxicity, and drug resistance.

Nanodelivery systems have demonstrated significant potential for the diagnosis and treatment of breast cancer bone metastases. These systems can efficiently deliver therapeutic agents and diagnostic reagents, thereby improving targeting and efficacy. Various nanoparticles (NPs) have been developed as contrast agents for medical imaging, addressing inherent limitations of traditional imaging modalities. The utilization of nanoparticles, including magnetic nanoparticles, enhances imaging capabilities and allows for targeted imaging. Compared to conventional probes, nanoparticles offer numerous advantages, such as controllable physical properties, ease of surface modification, and extended circulation times. Additionally, they can be integrated into multimodal imaging and therapeutic approaches. Diverse imaging modalities—including MRI, CT, fluorescence imaging, and positron emission tomography (PET)—have been employed to track nanoparticles within the body and to obtain structural, functional, and molecular information about tumor regions, each differing in sensitivity, penetration depth, and image resolution [9]. To achieve the goals of treatment, diagnosis, and prevention of BCBM, a variety of compounds—including anticancer therapeutics, contrast agents, photodynamic agents, and photothermal materials—have been employed for targeted delivery to bone [10]. Nanotechnology in drug delivery systems is rapidly evolving, showing promising trends in continuous innovation. This review focuses primarily on recent advances in nanodelivery systems for BCBM, which may offer new avenues for research and significantly alter the current landscape of diagnosis and treatment for breast cancer bone metastasis.

## 2. The Delivery Process of Drug Delivery Systems in Tumor

### 2.1. Biological Process of Bone Metastasis

**Figure 1:**
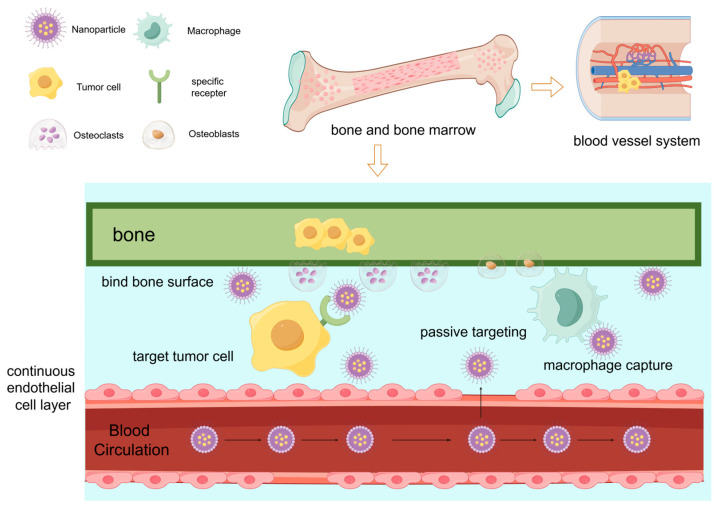
The nanoparticles through the blood–bone marrow barriers pathway (by Figdraw (https://www.figdraw.com)).

Osteocytes, the most abundant cells in bone tissue, regulate the activity of early cancer bone metastasis through interactions with both cancer cells and osteoclasts. Similar to other forms of distant metastasis, bone metastasis results from the interplay between metastatic cancer cells and the bone microenvironment, a phenomenon consistent with Paget’s “Seed and Soil” hypothesis proposed in 1889 [11] (the process of bone metastasis in breast cancer is shown in Figure 1). The bone microenvironment, metastatic tumor cells, osteoclasts, and tumor-associated macrophages (TAMs) collectively play critical and synergistic roles in establishing an environment conducive to the proliferation, progression, and survival of metastatic tumors, which ultimately induces osteoclast-mediated bone destruction.

The process of bone metastasis is complex, and numerous researchers have made strides in elucidating its underlying mechanisms. One key factor is the overexpression of receptor activator of nuclear factor κB ligand (RANKL) within the bone microenvironment, which creates a vicious cycle of bone metastasis and bone resorption [12]. Cadherin 11 (CDH11) serves as both an inducer and/or promoter of metastatic signaling in MBC, and it is a biomarker of poor prognosis. CDH11 is overexpressed at various favorable metastatic sites, including bone. Inhibition of CDH11 has been shown to reduce the migration and invasion capabilities of breast cancer cells, underscoring its critical role in the bone metastasis process. Notably, CDH11 modulates the classical Wnt signaling pathway through its interaction with β-catenin [13]. Furthermore, ectopic expression of miR-335 inhibits CDH11, while inhibition of miR-335 results in enhanced metastatic potential. In vivo studies indicate that administration of anti-CDH11 antibodies or miR-335 mimics can suppress tumorigenesis and inhibit cancer metastasis [14]. Tumors produce osteoclast-activating factors, and the resultant bone resorption promotes tumor cell growth, creating a “vicious cycle” of bone metastasis. Additionally, the relatively slow blood flow through the bone marrow and the presence of adhesion receptors on the bone marrow endothelium facilitate the localization of cancer cells within the bone. These features, combined with a bone marrow environment rich in growth factors and cytokines, further promote the progression of bone metastases [12].

### 2.2. Targeting Bone Marrow Metastasis Microenvironment

The microenvironment of distant organs is selectively altered by the primary tumor before metastasis [15,16]. The premetastatic niche is composed of a variety of complex factors [17]. Monotherapy may be insufficient to disrupt the premetastatic niche, and traditional treatments often have little effect on the premetastatic microenvironment. One study presented a nanotherapeutic approach based on enzyme-triggered transformation of polymers: after surgical resection of large primary tumors, this nanotherapeutic agent resides at the premetastatic niche and postsurgical wound sites, destroying the premetastatic microenvironment and eliminating micrometastases, fundamentally reducing metastasis and local–regional recurrence [18].

In the bone marrow metastatic microenvironment, different cell populations interact to regulate bone remodeling and hematopoiesis, processes that are essential for development, tissue regeneration, and immune function during disease progression. Once tumor cells enter this microenvironment, they must employ various strategies to survive and proliferate [19]. This insight offers a new avenue for the development of targeted therapies. By exploiting the distinct characteristics of tumor cells and the bone marrow metastatic microenvironment—compared to normal cells or other tumor targets—specific drug delivery systems can be designed to actively enrich therapeutic agents within both the tumor and the bone marrow metastasis microenvironment. Targeting the bone marrow metastasis microenvironment presents a complex but crucial therapeutic strategy. When designing targeting approaches, it is important to consider the coevolution of tumor cells and their microenvironment during metastatic progression to identify critical therapeutic windows. Specific components of the bone marrow microenvironment may serve as potential therapeutic targets for bone metastatic disease [20]. For example, therapeutic interventions targeting the metastatic niche by blocking the granulocyte-colony stimulating factor G-CSF receptor have demonstrated the potential to prevent pathological vascular remodeling and reduce the bone metastatic burden [21]. Another study developed nanoparticles containing doxorubicin (DOX)-loaded polylactic acid–hydroxyacetic acid (PLGA) and alendronate (ALN), which are nanoparticles capable of targeting the bone microenvironment, thereby reducing the number of osteoclasts and mitigating bone resorption in orthotopic mouse models of breast cancer translocation to bone [22]. The complexity of the tumor metastasis microenvironment continues to be a significant focus of research, with increasing attention from the scientific community.

In addition, the tumor microenvironment is often characterized by abnormal conditions, such as acidic pH, elevated reactive oxygen species (ROS), increased cytosolic glutathione, hypoxia, and overexpressed proteases. These factors can serve as endogenous triggers to stimulate the release of nanomedicines. Such microenvironmental characteristics are often taken into account in the design of nanomaterials to enhance the precision of their targeting capabilities. For instance, pH-sensitive nanoparticles developed by the Tao group for the codelivery of docetaxel (DTX) and dihydroartemisinin (DHA) represent a promising strategy for treating metastatic breast cancer via the ROS-mediated mitochondrial apoptosis pathway [23].

### 2.3. The Delivery Pathway of DDSs in Tumors

Nanoparticles, typically ranging from 100 to 500 nanometers in size, can be engineered to create intelligent drug delivery systems (DDSs) capable of encapsulating therapeutic agents and imaging agents. By manipulating factors such as size, morphology, surface modification, and material composition, nanostructures can be developed to provide controlled-release therapies and can help avoid premature clearance from the bloodstream, thereby maximizing circulation time. These targeted and sustained DDSs reduce drug-related toxicity and enhance patient adherence to treatment regimens [24,25]. DDSs can be tailored to deliver a variety of active substances, including antitumor drugs, small interfering RNA (siRNA), proteins, and contrast agents for the diagnosis and treatment of bone metastasis.

Tumor targeting using nanoparticles can be achieved through passive, active, or externally applied forces. In passive targeting, nanoparticles selectively accumulate at tumor sites due to the enhanced permeability and retention (EPR) effect. Tumor neovascularization is characterized by structural incompleteness and high permeability, which allows nanoparticles to penetrate the tumor microenvironment. The EPR effect, however, is more complex than initially understood and encompasses various biological processes, including angiogenesis, vascular permeability, hemodynamic regulation, the heterogeneity of the tumor’s genetic spectrum, and the tumor microenvironment, as well as lymphangiogenesis [26]. Active targeting, on the other hand, involves specific interactions between ligands on the surface of nanoparticles and biomarkers or receptors on target cells [27]. This approach can complement EPR-based passive targeting by enhancing the accumulation and retention of nanomedicines in tumors. A central concept in cancer nanomedicine is the use of nanoparticles to selectively accumulate at tumor sites [28]. Nanoparticles with high affinity selectively bind to targeted molecules expressed on the surface of cancer cells. Although functionalizing nanomaterials with targeting moieties may not always increase tumor accumulation, they generally enhance cellular uptake [28,29]. Following binding, nanoparticles are internalized by target cells via endocytosis. The molecular mechanisms underlying nanoparticle uptake are classified based on the size of the vesicles involved—phagocytosis for larger particles and pinocytosis for smaller ones [30].

In recent years, transcytosis could be an important positive factor for designing cancer nanomedicines, and transformable nanomedicine has become a new research hotspot to promote the penetration of nanomedicine in tumors. Transcytosis is a widespread process of transporting biomacromolecules across biological barriers. Through this process, substances are directly and actively transported from one side of a polarized cell to the opposite [31,32,33,34]. Transcytosis is an active behavior in cells that, due to its active chemotactic ability, can effectively target the transport of NPs, significantly enhancing the targeting effect [35]. Transcytosis can not only provide a driving force but also circumvent the inherent challenges of adverse microenvironments and the intrinsic size limitations of nanomedicine, and, thus, may facilitate the penetration of nanomedicine [32,36]. Meanwhile, enhanced tumor penetration promotes the therapeutic efficacy of a photodynamic therapeutic nanomedicine [32]. Transcytosis has been proposed as a potential active transport route for NPs, providing an alternative to the passive diffusion mechanism [37]. A study developed a transcytosable peptide-paclitaxel prodrug nanoparticle, which can be rapidly internalized by elial or tumor cells via receptor and adsorption-mediated transcytosis, enhancing the extravasation efficiency of tumor vessels and increasing the permeability of tumor tissues [38]. Tissue-targeted delivery, precise drug release at specific locations, and deep tissue penetration can be achieved by incorporating stimuli-responsive elements for transcytosis induction into the design of nanomedicines. Zhang et al. developed a small morph nanoparticle based on caveolae-mediated transcytosis that undergoes a rapid charge reversal from negative positive in response to the acidic conditions of the tumor microenvironment, enhancing its uptake by tumor cells and deep penetration into the tumor tissue both in vitro and in vivo [39]. A series of research findings emphasized the promising prospects of transcytosis-induced nanomedicine in tumor treatment [36].

Over the years, a variety of biochemical and biophysical tools have been developed to elucidate the intracellular trafficking properties, interacting moieties, subcellular localization, and spatiotemporal dynamics of nanoparticles within cells and organelles. Research continues to show that delivery efficacy depends on parameters such as uptake efficiency, retention, payload stability, endosomal escape efficiency, and subcellular localization [40]. Beyond their ability to target tumors and prolong circulation time in vivo, nanoparticles can also improve the immunosuppressive tumor microenvironment and activate tumor-killing T cells, thereby enhancing therapeutic efficacy while reducing the dosage and toxic side effects associated with treatment.

### 2.4. Characteristics of DDSs in Patients with Bone Metastasis

Specially designed nanoparticles can also serve as contrast agents for cancer diagnosis, facilitating high-sensitivity and high-resolution imaging for tumor detection. Furthermore, novel methods of tumor labeling and detection may be realized through the utilization of nanoprobes and nanobiosensors. By rationally designing and fabricating nanodrug carriers, the goals of targeted drug delivery in cancer therapy can be achieved. Additionally, nanoparticles can be employed for radiation sensitization and photothermal therapy to improve the treatment of malignant tumors [41]. DDSs effectively integrate diagnosis and treatment strategies for bone metastasis. However, challenges such as drug penetration barriers must still be addressed.

The occurrence of bone metastasis is closely associated to the unique structure of bone. The sinusoidal microcirculation structure on the surface of trabecular bone provides an optimal metastatic pathway for tumor cells [42]. Extracellular matrix (ECM) homeostasis is essential for organ development and function under physiological conditions, and ongoing modification or dysregulation of the ECM can contribute to pathological conditions. Cells involved in forming metastatic niches, such as cancer-associated fibroblasts (CAFs) and TAMs, play crucial roles in ECM alterations that promote cancer cell adhesion and growth [43]. For the effective application of DDSs, it is critical to consider not only the ECM environment but also the barriers to delivery. The vasculature between the bone marrow and the peripheral circulation forms a thin functional barrier known as the bone marrow–blood barrier, which consists of sinuses lined by a continuous layer of endothelial cells and a discontinuous outer membrane of reticular cells [44]. The crossing of the blood–bone marrow barrier represents an initial and essential step in the process of nanoparticle delivery and action [45,46]. In Figure 1, we illustrate how nanomedicine can traverse the blood–bone marrow barrier. To efficiently target mineralized bone tissue, systemic DDSs must pass through the blood–bone marrow barrier, which consists of capillary fissures in the bone marrow sinus with diameters of approximately 80–100 nm. Given the low permeability of this barrier, the size of nanoparticles is a key factor in their ability to permeate it [47]. Therefore, the unique characteristics of bone metastasis must be fully considered during the development and preparation of a DDS.

## 3. The Diagnosis and Monitoring of Drug Delivery Systems in Breast Cancer Bone Metastasis

### 3.1. Drug Delivery Systems for Imaging and Early Detection

Detecting bone metastases in breast cancer at an early stage can be challenging due to the small and often multiple nature of the lesions. Nanotechnology presents a promising strategy in preclinical research, offering the ability to identify primary tumors and metastases early. This multifunctional, controllable, and traceable approach could revolutionize cancer research and treatment, particularly in imaging and DDSs [48] (relevant studies for imaging are shown in Table 1). To date, various nanoparticles (NPs) have been explored for molecular imaging and studied extensively in fluorescence imaging and MRI.

- Applications of imaging

While MRI is a widely used noninvasive diagnostic tool, conventional contrast agents tend to be toxic, nonspecific, incapable of penetrating biological barriers, and are excreted quickly. Gadolinium-based chelates are commonly used in clinical MRI, but their toxicity and potential persistence in the body raise safety concerns. The Food and Drug Administration has issued warnings related to these risks [49]. However, encapsulating contrast agents in nanocarriers can mitigate many of these problems, and leveraging the inherent properties of DDS carriers offers further advantages [50]. Iron oxide nanoparticles (IONPs) are seen as an attractive alternative due to their nontoxic and biodegradable properties [49]. For example, researchers have coloaded superparamagnetic iron oxide nanoparticles (SPIONs) with the chemotherapeutic drug DOX into PLGA nanoparticles targeting the AS1411 aptamer. This formulation improves the contrast in magnetic resonance images of tumor sites [51]. However, diagnosing small metastases early remains crucial to treating stage IV breast cancer effectively. In studies with transgenic mouse models, researchers labeled SPIONs coated with chitosan and polyethylene glycol (PEG) copolymers with fluorescent dyes and targeted the neu receptor with a monoclonal antibody (NP-neu). This nanosystem significantly enhanced contrast in primary breast tumor images and demonstrated the potential to detect micrometastases via MRI [52]. The CD44 protein is closely associated with tumor growth, proliferation, metastasis, invasion, and angiogenesis. Scientists have developed HA-modified magnetic nanoclusters to detect CD44-overexpressing breast cancers using MRI. These nanoclusters were synthesized by conjugating pyrenyl hyaluronan (Py-HA) as CD44-targetable surfactants to hydrophobic magnetic nanocrystals. In vitro and in vivo studies demonstrated excellent targeting efficiency, sensitivity, and biocompatibility. Additionally, HA-modified magnetic NPs successfully illuminated tumors on MRI, suggesting their potential as a molecular imaging agent for CD44-targeted tumor detection [53].

Radionuclide imaging has also been employed to image radiolabeled NPs in metastatic breast cancer. Animal and histological studies showed that using low doses of nanoparticles with radionuclide imaging enhances the precise targeting of micrometastases in a 4T1 breast cancer mouse model. Gold nanoparticles, labeled with 99mTc and αvβ3-targeting ligands, were used to visualize micrometastases at low doses using radionuclide imaging [54].

Near-infrared (NIR) imaging has also shown promise for imaging both primary and metastatic cancers. NPs or NIR light emission from NPs conjugated with NIR dyes have been used for lymph node metastasis imaging in mouse models. Lipid/calcium/phosphate NPs, around 25 nm in size, have demonstrated the ability to infiltrate mouse tissues and accumulate in lymph nodes via lymphatic drainage. In a 4T1 breast cancer lymph node metastasis model, intravenously administered 111In-lCP demonstrated sentinel lymph node tumor enlargement [55]. Combining MRI with high-sensitivity imaging modalities, such as near-infrared fluorescence (NIRF) and PET, and utilizing multimodality imaging contrast agents, has significantly reduced data processing time while improving diagnostic accuracy [56]. Researchers have also created nano-hybrid ceramide liposome nanoparticles loaded with paclitaxel (PTX), which were modified with programmed death ligand-1 (PD-L1) antibodies for specific targeting. After imaging with NIR and MRI, these intravenously administered nanoparticles showed a strong tumor-positive contrast. Compared to nontargeted PD-L1 antibodies and paclitaxel delivery, this system was more effective in targeting tumors and metastases. The results suggest that dual-labeled ceramides represent a promising therapeutic agent for MRI/NIRF dual-modality detection and in situ treatment of solid tumors [57].

- Multimodal imaging technologies

To overcome the limitations of single imaging modalities, multimodal imaging technologies have been developed. For example, magnetic liposomes have shown promise as efficient multimodal contrast agents. These liposomes not only provide contrast but also enhance the accumulation of nanoparticles at targeted sites, offering improved bioavailability. NP contrast agents for MRI have advantages over conventional contrast agents, including higher sensitivity and longer blood circulation times [58]. However, T2 contrast MRI (dark contrast imaging) poses challenges, as it can be difficult to distinguish from artifact signals related to internal hemorrhage, calcification, metallic deposits, or air–tissue boundaries. Consequently, researchers have sought to improve diagnostic efficiency by using T1 MRI and multimodal imaging [59]. The development of integrated multimodality contrast agents (MCAs) for MRI/CT multimodality imaging is gaining traction in current research. A team designed a novel MCA (F-AuNC@Fe_3_O_4_) by assembling gold nanocages (Au NC) with ultrasmall iron oxide nanoparticles (Fe_3_O_4_) for simultaneous T1-T2 dual MRI and CT contrast imaging. In this nanodrug delivery system, the gold nanocages provide simple thiol modification and strong X-ray attenuation for CT imaging, while the ultrasmall Fe_3_O_4_ nanoparticles serve as excellent contrast agents for both T1 and T2 weighted MRI. The functionalized MCA nanoparticles exhibit a small average particle size, low aggregation, and good biocompatibility [60]. Functional nanoparticles, such as glucose-coupled nanoparticles, show promise for being taken up by cancer cells at a higher rate and could be developed as imaging agents for the early diagnosis of breast cancer. For instance, gold-coated silicon dioxide nanoparticles have been used as multimodal contrast agents in fluorescence, magnetic resonance, and photoacoustic tomography [61]. Functional nanoparticles, such as glucose-coupled nanoparticles, show promise for being taken up by cancer cells at a higher rate and could be developed as imaging agents for the early diagnosis of breast cancer. For instance, gold-coated silicon dioxide nanoparticles have been used as multimodal contrast agents in fluorescence, magnetic resonance, and photoacoustic tomography [5]. An NIF dye, IR825, is adsorbed on serum albumin acid (HSA), which is covalently linked to diethylenetriamine pentaacetic acid molecules to sequester gadolinium. The formed HSA-Gd-IR825 nanocomposite has strong fluorescence and high near-infrared absorption, and can be used as a T1 contrast agent for magnetic resonance imaging. Dual-mode fluorescence and imaging in vivo revealed that HSA-Gd-IR825, after injection into the primary tumor, rapidly migrates into the tumor-associated sentinel lymph node via the lymphatic circulation. The strong near-infrared absorption of HSA-Gd-IR825 can effectively ablate the sentinel lymph node with metastatic cancer cells under the irradiation of the near-infrared laser. Albumin-based therapeutic nanoprobes, with multimodal imaging and photothermal therapy capabilities, as well as a “photothermal ablation-assisted surgery” strategy, hold promise for clinical cancer therapy in the future [62].

- Imaging combined with multiple treatment methods

Therapeutic W_18_O_49_ nanoparticles targeting human epidermal growth receptor 2 (HER-2) overexpression in breast cancer were synthesized by the polyol method. With high X-ray attenuation and photothermal efficacy, the lymph nodes of HER-2-positive metastatic mice can be clearly imaged and recognized under CT guidance, and the lymph nodes can be selectively cleared by laser ablation [63]. The team self-assembled three clinically approved drugs, including serum albumin, paclitaxel, and indocyanine green, into a new Abraxane-like nanocomposite. In this study, the combination of photothermal and chemotherapy using imaging-guided therapy not only effectively eliminated subcutaneous tumors, but also significantly inhibited the development of metastatic tumors. Indocyanine green induced by near-infrared laser irradiation and moderate photothermal heating promoted the intracellular uptake of NPs to improve the killing of cancer cells [64]. Researchers produced a tumor-specific pH-reactive peptide H7K (r 2)2-modified therapeutic liposome PTX and superparamagnetic iron oxide nanoparticles (SPIO NPs) PTX/SPIO-SSL-H7K (r 2)2; among them, H7K (R2)2 was the target ligand, SPIO NPs was the magnetic resonance imaging agent, and PTX was the antitumor drug. The pH response of H7K (R2)2 in the MDA-MB-231 cell line was confirmed in vitro and in vivo. The targeted modified therapeutic liposomes can achieve the dual effects of antitumor and MRI imaging [65].

Multimodal imaging is the research hotspot in the field of imaging, and many exciting results have been achieved in recent years. Multimodality imaging combined with various therapeutic methods has also achieved good results in experiments. By combining NIR-II FL/PA dual-mode imaging with photothermal immunotherapy in one component, researchers have designed a novel integrated nanotherapy system to precisely localize the microlesion of bone metastasis and achieve complete tumor ablation in the early stage. Surface modification with ibandronate is beneficial for passive and active targeting, and can significantly improve the detection rate of bone metastasis and inhibit bone resorption. Superior photothermal properties generate enough heat to kill tumor cells while stimulating upregulation of heat shock protein 70, which triggers immunogenic cell death (ICD-RRB-effects and antitumor immune responses. These integrated nanosystems accurately demonstrate the localization of early lesions in bone metastasis and whole-tumor ablation through a single integration of “single-component, multifunctional” technologies [66]. Multimodal tumor-cell-derived extracellular vesicles (TEVs)-based nanoplatforms are used for multimodal miRNA delivery and phototherapy, as well as cancer magnetic resonance imaging. TEV as a bionic source of gold-iron oxide nanoparticles (GIONs) was investigated, and the thermal properties of TEV-GIONs were demonstrated in vitro. The results suggest that the distribution pattern of TEV-anti-miR-21-GIONs is well correlated with tumor-targeting ability, activity, and efficacy obtained in response to doxorubicin combination therapy. The applications of TEV and TEV–GIONs in molecular imaging and therapy of cancer are promising [67].

Nanotechnology has shown tremendous advantages over traditional methods in targeting and imaging breast cancer metastasis. Nanotechnology-based drug delivery systems offer considerable advantages over traditional imaging and detection methods, including stronger cell–cell interactions, higher imaging sensitivity and resolution, longer blood circulation times, and lower toxicity. These systems have great potential for development and application in tumor imaging, particularly for breast cancer metastasis detection and treatment.

### 3.2. Research Progress of Drug Delivery Systems in Imaging of Breast Cancer Bone Metastasis

To address the limitations associated with bone metastasis imaging in breast cancer, researchers have increasingly focused on nanodrug delivery systems to enhance early diagnostic capabilities and improve the accuracy of detecting multiple metastases, while also aiming to reduce adverse drug effects. Especially, several MRI-traceable tumor-targeted delivery systems have been developed to overcome the complex barriers of drug delivery in different types of cancer, providing real-time assessment of treatment response. Pourtau, L. et al. developed a new MCA for bone metastasis imaging, using a multifunctional polymer loaded with magnetite nanoparticles and grafted with antibodies targeting the human endothelial receptor 2. After administration to mice bearing bone tumors grown from human breast cancer cells, images showed retention of antibody-labeled polymers that targeted and enhanced the tumor site. This provides a positive signal for targeted imaging in BCBM [68]. Another innovative approach involved the creation of a pH-responsive bone-targeting drug delivery system using zoledronic acid–anchored bimodal mesoporous silica to encapsulate gadolinium (III) upconversion nanoparticles loaded with plumbagin. This platform enables more sensitive and specific detection, as well as treatment of bone metastases in their early stages. Such advances highlight the growing importance of nanotechnology in enhancing the diagnosis and treatment of BCBM [69]. Research into nanotechnology-based imaging for BCBM continues to grow, with increasing efforts to integrate diagnosis and therapy. Dual-targeting approaches for osteoclasts and tumor cells have been developed using biomimetic semiconductor polymer nanocomposites (SPFeNOCs). This system contains a hybrid membrane of semiconductor polymers and iron oxide (Fe_3_O_4_) nanoparticles, camouflaged with cancer and osteoclast cell surfaces. This unique design facilitates the homing and accumulation of nanoparticles in tumor sites via a homologous targeting mechanism, enabling the system to target both metastatic tumor cells and osteoclasts involved in bone metastasis. In addition to its targeting ability, the semiconductor polymer facilitates NIRF imaging and sonodynamic therapy (SDT), while Fe_3_O_4_ nanoparticles enable MRI and chemodynamic therapy (CDT). This nanosystem not only effectively detects bone metastases from 4T1 breast cancer but also disrupts the vicious cycle of bone metastasis to achieve a high level of antitumor activity [70]. These exciting findings in nanodrug delivery systems for targeted imaging in BCBM have encouraged more researchers to invest in this field. The ongoing innovation and application of high-precision diagnostic models are expected to improve the diagnosis and treatment of breast cancer metastases significantly.

## 4. The Progress of Drug Delivery Systems in the Treatment of Bone Metastasis of Breast Cancer

**Figure 2:**
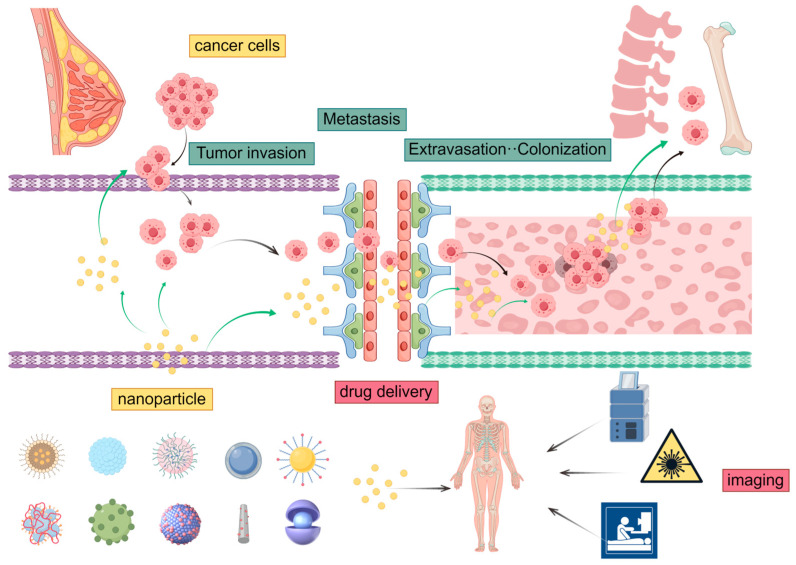
The process of bone metastasis in breast cancer and the mode of action of drug delivery systems (by Figdraw).

Advanced imaging techniques have significantly improved the detection of various types of metastatic breast cancer. NPs loaded with therapeutic drugs and modified with target ligands are widely used in treatments such as chemotherapy, targeted therapy, photothermal therapy, and combination therapies. Currently, numerous nanomaterials, including liposomes, polymeric micelles, polymeric nanoparticles, dendrimers, carbon nanotubes, and graphene oxide, are utilized in the treatment of bone metastasis. These advanced delivery systems play a crucial role in enhancing therapeutic efficacy and monitoring disease progression (the mode of action of the drug delivery system is shown in Figure 2).

### 4.1. Cancer Drug Treatment Based on Drug Delivery Systems

In BCBM, common molecular targets include hydroxyapatite (HA) and αvβ3 integrin. DDSs targeting αvβ3 integrin for the delivery of drugs like docetaxel have shown promising results, demonstrating reduced bone destruction, lower liver toxicity, and decreased tumor burden compared to free docetaxel at equivalent doses [71,72]. One study combined curcumin and bortezomib in PLGA nanoparticles. The aluminum-coated nanoparticles reduced bone resorption and tumor growth in an intraosseous metastasis model, illustrating how ALN can efficiently adhere to nanoparticle surfaces to deliver multiple therapeutic agents into the bone microenvironment. Bisphosphonate-modified NPs have shown enhanced bone binding and uptake in both in vitro and in vivo studies, including those using breast cancer cell lines (MDA-MB-231), demonstrating high bone marrow accumulation [73]. Researchers made polyethylene glycol (PEG)-coated NPs, which linked a Zn^2+^coordination polymer with ALN, to deliver cisplatin prodrug to the bone. The nanosystem not only significantly reduces cisplatin’s toxicity, but also inhibits tumor growth and reduces bone fragmentation [74]. Another study comprised poly PLGA as the hydrophobic core coated with alendronate-modified D-α-tocopherol polyethylene glycol succinate and developed folic acid-conjugated NP as a vehicle for PTX. The bone destruction and bone loss in tumor-bearing mice were significantly delayed after treatment, which showed potential therapeutic effects [75].

Zoledronate (ZOL), a third-generation bisphosphonate, is commonly used in treating bone metastasis due to its strong affinity for bone tissue. Recent studies have focused on combining ZOL with other drugs to create functionalized drug delivery systems. For instance, metal–organic framework (MOF) nanoparticles loaded with cytosine–phosphate–guanosine (CpG) and surface-modified with ZOL showed promise in inhibiting osteoclast-mediated bone destruction while enhancing M1 macrophage polarization [76]. Combining ZOL with chemotherapy drugs such as docetaxel has demonstrated a synergistic effect, reducing the necessary dosage of less-selective chemotherapy agents and improving therapeutic outcomes with fewer side effects [77]. Another innovative approach involves ZOL-modified PLGA nanoparticles. Compared to pegylated PLGA nanoparticles, these ZOL-modified systems showed improved cellular uptake and higher cytotoxicity in MCF-7 and BO_2_ cell lines. In vitro and in vivo studies demonstrated that these nanoparticles had better biodistribution, inhibited primary tumors and bone metastases, and reduced bone erosion [78]. Elham Hatami reported a novel nanoparticle composed of poly-(vinylpyrrolidone) and a tannic acid core, which forms a self-assembly with zoledronic acid. This nanoparticle demonstrated enhanced delivery of zoledronic acid to tumor cells and, importantly, sensitized the cancer cells to treatment with docetaxel [79].

To enhance the therapeutic effect of DOX on bone metastasis, researchers developed multifunctional micelles that combine pH sensitivity with bone-targeting capabilities. Doxorubicin–polyethylene glycol–alendronate (DOX-hyd-PEG-ALN) micelles delayed tumor growth, reduced bone loss, and mitigated cardiac toxicity compared to free DOX [80]. Liposomes modified with ALN and low-molecular-weight heparin have similarly shown significant tumor growth inhibition and metastasis reduction [81]. Responsive DDSs designed to adapt to environmental stimuli, such as pH or redox conditions, are another area of innovation. A pH- and redox-sensitive DDS (DOX@ALN-(HA-PASP) CL) has demonstrated GSH- and pH-dependent DOX release. In a 3D breast cancer bone metastasis model, this system inhibited both the proliferation of MDA-MB-231 cells and osteoclast activity. In vivo studies showed that the system significantly reduced tumor volume and bone resorption in tumor-bearing mice without causing systemic toxicity [82]. Calcium phosphosilicate nanoparticles encapsulated with gemcitabine monophosphate have the potential to be used as imaging tools and selective drug delivery systems for BCBM [83].

For the treatment of triple-negative breast cancer, C-X-C chemokine receptor type 4 and docetaxel (DTX) have been incorporated into DDSs to inhibit bone-specific and lung-specific metastases [84]. The encapsulation of DTX in ALN-modified DDSs has similarly shown promise in inhibiting tumor growth and treating bone metastases [85,86].

In addition to delivering conventional bone-targeting agents and chemotherapy, numerous studies are constantly seeking alternative therapies to reduce tumor-related bone destruction. One study developed a micellar NP to encapsulate and colloidally stabilize GANT58, a Gli antagonist, providing a fully aqueous, intravenously injectable formulation based on the polymer. The results showed that in the tibia model of BCBM, GANT58-NPs treatment reduced the area of bone lesions by 49%, reduced the number of lesions by 38%, and increased the volume of bone trabeculae by 2.5 times [87]. Another study designed a bone-targeted nanoparticle comprising an amphiphilic diblock copolymer of poly-[(propylene sulfide)-block-(alendronate acrylamide-co-N,N-dimethylacrylamide)] [PPS-b-P(Aln-co-DMA)] to encapsulate and preferentially deliver GANT58. This formulation may effectively inhibit tumor-driven osteoclast activation and the resulting bone destruction in patients with bone-related tumor metastases [88].

Researchers are continuously exploring combinations of different drugs, nanomaterials, and targeting pathways to optimize DDSs for BCBM therapy. These efforts aim to develop systems that offer high efficacy, minimal side effects, and broad adaptability across different patient populations.

### 4.2. Photodynamic and Photothermal Therapy Based on Drug Delivery Systems

Phototherapy is a fast-developing cancer therapy that uses various wavelengths of light to induce photochemical or photothermal changes in target tissues. The two most common phototherapies include photodynamic (PDT) and photothermal (PTT) therapy, which utilize light and exogenous or endogenous absorbents to generate cytotoxic ROS or local temperature rise, respectively [89].

PTT employs light-absorbing materials to convert absorbed photon energy into heat, effectively killing cancer cells when combined with NIR lasers. One study reported a dual-targeted and photothermally triggered nanotherapeutic system based on superparamagnetic iron oxide (Fe_3_O_4_) and indocyanine green (ICG)-embedded poly-LRB-lactide) copolymer (PLGA-ZNPS NICG(ICG/Fe_3_O_4_@PLGA-ZOL) PTT for tibia metastasis of breast cancer. Both ICG and Fe_3_O_4_ convert light into heat, while Fe_3_O_4_ and ZOL nanoparticles can be attracted to specific sites in the bone by an external magnetic field. Dual-targeting and dual-photothermic agents ensure high aggregation in the tibia and perfect PTT efficiency [90]. Shuo Jie et al. developed a novel oxygen-vacancy-rich tungsten bronze nanoparticle (Na_x_WO_3_) through a simple pyrogenic decomposition process for PTT. In both in vitro and in vivo experiments, the synthesized Na_x_WO_3_ nanoparticles exhibited excellent PTT ability and a potent tumor ablation effect in breast-cancer-induced osteolytic bone metastasis [91]. Another produced bioactive multifunctional CePO4/chitosan (CS)/graphene oxide (GO) scaffold, a promising platform for the treatment of bone metastases from breast cancer. Go-modified CEPO4 nanorods can effectively treat bone metastasis of breast cancer and improve bone regeneration. This multifunctional CEPO4/CS/GO scaffold holds great potential as a treatment platform for bone metastasis [92]. Additionally, a photothermal-triggered nanomaterial based on IR780-embedded PLGA nanoparticles (IR780@PLGA NPs) has been reported for the PTT treatment of breast cancer with bone metastasis. IR780 acts by converting light into heat, effectively “burning” the tumors. This nanosystem demonstrated impressive results in the PTT-based treatment of bone metastases [93].

PDT, a noninvasive, safe, and selective therapy, has been extensively studied for the treatment of various cancers. By utilizing photosensitizers, appropriate wavelengths, and molecular oxygen, PDT generates ROS, leading to cell necrosis or apoptosis. Clinically approved as a minimally invasive therapy, PDT shows selective cytotoxic activity against malignant cells. This method involves applying a photosensitizer followed by irradiation at a wavelength corresponding to its absorption band. In the presence of oxygen, a series of reactions occurs, resulting in direct tumor cell death, damage to microvessels, and local inflammatory responses. PDT boasts minimal toxicity to normal tissues, negligible systemic effects, significantly reduced long-term morbidity, a lack of intrinsic or acquired resistance mechanisms, and excellent preservation of cosmetic and organ function, making it a valuable option for combination therapy [94]. One study constructed ALN-functionalized bone-seeking nanoparticles (BTZ@ZnPc-ALN) to codeliver the proteasome inhibitor bortezomib (BTZ) and the photosensitizer zinc phthalocyanine (ZnPc) for synergistic chemical photodynamic therapy of bone metastases. BTZ@ZnPc-ALN has good bone affinity in vitro and in vivo, and can release drugs in a pH-responsive manner. Under irradiation, BTZ@znpc-ALN can produce ROS that cause mitochondrial damage and increase cytosolic Ca^2+^ levels and GRP78 protein expression, inducing excessive endoplasmic reticulum stress that synergistically inhibits cell proliferation [95]. Additionally, based on liquid metal nanoparticles, a new strategy for treating bone metastasis and reducing bone resorption was proposed, leveraging autophagy activation to counteract thermal resistance induced by mild PTT. This model has yielded promising results [96]. These constant innovations offer new perspectives and pave the way for further exploration in the field.

### 4.3. Other Treatments Based on Drug Delivery Systems

Innovative therapeutic approaches for MBC are continuously emerging, and new strategies such as gene therapy and immunotherapy have shown encouraging results. The integration of nanotechnology with these therapies offers significant potential, enabling more precise and effective treatments that address the limitations of conventional therapies. Traditional gene therapy methods, like the use of free nucleic acids, face challenges due to poor cellular uptake and instability in circulation. Rapid developments in the field of nanomedicine aimed at reducing the delivery of targeted drugs/genes for BC are expected to overcome the limitations of traditional therapies. Nanodelivery platforms can be effective carriers of specific drugs/genes by improving cycle time, improving bioavailability, reducing the chance of recognition by the immune system, and accurately delivering gene regulators, and can change the situation of BC gene therapy. SiRNA is a promising gene-silencing tool because it can specifically inhibit cancer-associated genes and help maintain homeostasis between osteoclasts and osteoblasts. In one study, siRNA was loaded onto a targeted E-selectin thioaptamer-conjugated multistage vector. The therapeutic effect of siRNA on breast cancer bone metastasis was evaluated in a mouse xenograft model of MDA-MB-231 breast tumors, and the results demonstrated effective inhibition of intraosseous tumor growth [97].

The immunosuppressive microenvironment within bone metastases limits the effectiveness of immunotherapy, leading to poor outcomes in large patient populations. Nanoparticle-based approaches are being developed to regulate the antitumor immune response and improve treatment outcomes. For example, immunostimulants loaded onto nanoparticles can be specifically delivered to the tumor microenvironment, enhancing local immune responses. This approach shows promise for overcoming tumor immune evasion and increasing tumor rejection [98]. Immunostimulants can be loaded onto NPs and delivered specifically to the tumor microenvironment to activate local immune responses. Nanodrugs provide a promising opportunity to improve the efficacy of cancer immunotherapy by improving the delivery, retention, and release of immunostimulants in target cells and tumor tissues. Therefore, they can be used in breast cancer to overcome tumor immune escape and increase tumor rejection [99]. Persistent hypoxia in bone metastases induces an immunosuppressive environment that limits the effectiveness of immunotherapy. To address chronic hypoxia, the researchers developed manganese dioxide (MnO) nanoparticles with adjustable oxygen generation kinetics for persistent oxidation of metastatic bone lesions. Sustained control of hypoxia using PEG-stabilized MnO_2_ or poly-LRB-lactic-co-glycolic acid) enhances the cytotoxicity of natural killer cells to tumor spheres; it also shows a strong build-up in the long bones and pelvis, a common site of bone metastases. The nanoparticles improved the survival of mice with established bone metastases by reducing hypoxia and regulatory T cell levels in the tumors [100].

### 4.4. Multimodality Combined Therapy Based on Drug Delivery Systems

DDSs are not limited to the delivery of a single therapeutic agent. They can also integrate multiple treatment modalities, providing synergistic therapeutic effects. Several studies have demonstrated the success of combination therapy models for treating bone metastases from breast cancer.

For example, a bone-targeting nanoplatform was developed by encapsulating gold nanorods in mesoporous silica nanoparticles (Au@MSN), which were then combined with ZOL. This multifunctional nanoparticle system not only demonstrated bone-targeting properties but also inhibited osteoclast-like cell formation, promoted osteoblast differentiation, and, when triggered by near-infrared irradiation, inhibited tumor growth and reduced bone resorption [101]. Another study focused on a multifunctional nanoplatform that combined Gd_2_O_3_ nanoparticles with near-infrared light-absorbing polymer polypyrrole (PPy), HA, and aluminum phthalocyanine (ALPC). These Gd_2_O_3_@PPy/ALPC-HA nanoparticles were used for fluorescence (FL)/MR/photoacoustic (PA) imaging-guided PTT and PDT. In vivo studies showed enhanced tumor uptake of these nanoparticles and superior antitumor effects compared to single-therapy approaches [59]. A further example involved engineered macrophages (Oxa (IV)@ZnPc@M), which were designed to carry nanodrugs containing oxaliplatin prodrugs and photosensitizers. These NIR light-activated drug carriers demonstrated enhanced chemotherapy, PDT, and immunotherapy for primary and bone metastases. The combined therapy effectively eliminated tumors while activating tumor-specific antitumor immune responses [102]. The combination of multiple therapeutic modalities using DDSs presents a promising strategy for developing more effective treatment plans for breast cancer bone metastasis. This multimodality approach paves the way for further exploration in the field of cancer therapy.

## 5. Conclusions

At present, there is no standardized or highly effective treatment for BCBM. Most therapies for bone metastases from breast cancer are primarily used for palliative purposes, with emphasis on pain management, reducing the risk of bone-related complications, and inhibiting tumor progression. Nanodelivery systems present a groundbreaking approach and hold significant promise for the diagnosis and treatment of BCBM, addressing many limitations of traditional therapies. Based on many preclinical studies, DDSs have shown great advantages in the field of imaging. It is possible to achieve early detection of bone metastases in the breast cancer population and achieve radiographic predictive power. Additionally, the integration of diagnosis and treatment may become feasible through the use of nanomaterials, offering a combined diagnostic and therapeutic model for BCBM. By enhancing drug bioavailability, reducing immune recognition, and enabling targeted delivery, these systems improve the efficacy of gene therapy, immunotherapy, and combination therapies. Significant advancements, such as siRNA-loaded vectors for gene silencing and nanoparticle-based immunomodulation, highlight their potential to overcome tumor resistance and increase treatment precision. Additionally, multimodality approaches combining photothermal therapy, photodynamic therapy, and chemotherapy show promising results in enhancing therapeutic outcomes.

However, challenges remain, including the complexity of system design, clinical translation barriers, and the need for more comprehensive studies on toxicity and pharmacokinetics. Although significant progress has been made, many challenges remain before these systems can be widely adopted in clinical practice. Future research should focus on refining nanodelivery systems, exploring innovative materials, and ensuring safety and efficacy in clinical applications. Ultimately, these efforts will pave the way for personalized medicine in the treatment of BCBM.

## 6. Future Development Prospects

- Challenges and Gaps

Despite significant progress in the development of MRI-targeted nanotherapy diagnostic platforms, substantial knowledge gaps continue to hinder the transition from the research bench to the bedside. Few nanotherapy diagnostic systems have progressed through clinical trials, despite their potential in prediction, prevention, and personalized medicine.

The complexity of nanodelivery systems, difficulties in predicting interactions with biological systems, immune responses, and issues with toxicity have delayed clinical trials. Additionally, the pharmacokinetics and biodistribution of these systems are difficult to control, with the risk of premature release of therapeutic agents in the bloodstream and healthy tissues. Studies indicate that over 85% of reported stealth nanomaterials exhibit a rapid decrease in blood concentrations to half their initial dose within one hour of administration, despite the presence of a relatively long β-phase. While the stealth effect and pseudo-stealth effect may improve pharmacokinetics, the dynamic modulation of the stealth properties of nanomaterials or DDSs remains a significant challenge, though not an insurmountable one. This field continues to be a major focus of academic research [103]. Moreover, the substantial differences between animal models and human patients further complicate the transition from bench to bedside [29].

- Research Needs

The basic research and application of nanodrug delivery systems for BCBM can be further deepened. New nanomaterials with higher therapeutic loading capacities and adjustable payload release curves should be developed. The expansion of research into innovative nano-DDSs, including material upgrades and improved drug delivery methods, is essential to enhance the accuracy of diagnosis and treatment. Additionally, the metabolism of nanomedicines and their effects on healthy bone remain unclear, limiting the clinical translation of bone-targeted NPs. Thus, studies on the metabolism and safety of bone-targeted NPs in bone tissue are critical. More advanced in vitro models of breast cancer metastases are also needed to evaluate the therapeutic efficacy of NPs. Furthermore, additional in vivo studies on efficacy and pharmacokinetics are essential for promoting the clinical use of these systems.

Currently, most nanomedicines remain in the in vitro research phase, with mass production yet to be achieved. Standardizing the application of nano-DDSs is challenging due to the high-level refinement required in nanotechnology and the complexity of the preparation process. Current limitations of nanodelivery systems for BCBM include the complexity of the synthesis process and poor reproducibility. Key experimental parameters for the mass production of nanoparticles, such as synthesis and conjugation processes, must be precisely controlled, which may require more stringent production standards and processing conditions.

- Future Directions

Continued research in nanotechnology is expected to contribute to a better understanding of the pathogenesis of breast cancer bone metastasis and to the development of more effective nanodrugs. By improving the integration of diagnosis and treatment and expanding the use of innovative nanodelivery systems, researchers can enhance the accuracy of both diagnostic and therapeutic approaches. With sustained efforts, nanomedicine may eventually bridge the gap between laboratory research and clinical practice, offering a promising future for the treatment of BCBM (Table 2).

## Figures and Tables

**Table 1:** Relevant studies for imaging.

Imaging Method	MCA	Imaging System
MRI	Iron oxide nanoparticles	SPIO/Dox-NPs
MRI	Iron oxide nanoparticles	SPIONs/PEG
MRI	Magnetic nanoparticles	HA-mnCs
Radionuclide imaging	Au nanoparticles	AuNP/αvβ3
NIR	ICG	ICG-NPs
MRI/CT	Au nanocages + ultra-small iron oxide nanoparticles	F-AuNC@Fe_3_O_4_
Fluorescence/MRI/PAT	Gold-speckled silica NPs	GSS
Fluorescence/CT/MRI	GdF_4_ + SiO_2_ + Au	NaY/GdF_4_: Yb,Er,Tm@SiO_2_-Au@PEG (5000)
Fluorescence/MRI	IR825	HSA-Gd-IR825
MRI	SPIO NPs	PTX/SPIO-SSL-H7K(R2)2
MRI	Gold–iron oxide NPs	TEV-anti-miR-21-GIONs

**Table 2:** Relevant studies for drug delivery systems in the treatment of BCBM.

Treatment Method	Payload	Delivery System	Reference
Drug therapy	ALN	PLGA-NPs	[60]
	ALN	PEG-NPs	[61]
	ALN/PTX	PLGA-NPs	[62]
	ZOL	MOF-NPs	[63]
	ZOL	PLGA-NPs	[65]
	Zoledronic acid	Poly(vinylpyrrolidone)-NPs	[66]
	DOX/ALN	PEG-micelles	[67]
	ALN	Liposomes	[68]
	DTX/ALN	Polymeric micelles	[72]
Photothermal therapy	ICG/Fe_3_O_4_-ZOL	PLGA-NPs	[77]
	Na_x_WO_3_	NPs	[78]
	CePO_4_-GO	CePO_4_/CS/GO scaffold	[79]
	IR[780]	PLGA-NPs	[80]
Photodynamic therapy	BTZ@ZnPc-ALN	NPs	[81]
	Liquid metal	Liquid metal NPs	[83]
Gene therapy	siRNA	Thioaptamer	[84]
Immunotherapy	MnO_2_	PEG-NPs	[87]
Multimodality therapy	Gold nanorods/ZOL	Multifunctional NPs	[88]
	Gd_2_O_3_@PPy/ALPC-HA	Multifunctional nanoplatform	[46]
	Oxaliplatin prodrugs/photosensitizers	Nanodrugs	[89]
